# Trends and Characteristics of Young Non-Small Cell Lung Cancer Patients in the United States

**DOI:** 10.3389/fonc.2015.00113

**Published:** 2015-05-26

**Authors:** Anish Thomas, Yuanbin Chen, Tinghui Yu, Marko Jakopovic, Giuseppe Giaccone

**Affiliations:** ^1^Medical Oncology Branch, National Cancer Institute, National Institutes of Health, Bethesda, MD, USA; ^2^Office of Surveillance and Biometrics, Devices and Radiological Health, Federal Drug Administration, Silver Spring, MD, USA; ^3^Department for Respiratory Diseases, School of Medicine, University Hospital Center Zagreb, University of Zagreb, Zagreb, Croatia; ^4^Lombardi Cancer Center, Georgetown University, Washington DC, USA

**Keywords:** non-small cell lung cancer, SEER, young, population-based studies, disparities

## Abstract

**Background:**

Although the median age at diagnosis of non-small cell lung cancer (NSCLC) is 70 years, a subset of patients with NSCLC present at a younger age (<40 years). Little is known about the time-trends in incidence of NSCLC in the young, their characteristics and outcomes.

**Methods:**

The surveillance, epidemiology, and end results database was used to extract NSCLC cases from 1978 to 2010. Yearly incidence rates in various age groups, race, site of disease, histology, treatment patterns, and outcomes were assessed. We modeled Kaplan–Meyer survival curves stratified by age of presentation.

**Results:**

Young patients represented 0.6% of incident NSCLC from 1978 to 2010. The incidence of young NSCLC declined significantly during this time-period. Young NSCLCs had a higher proportion of women (51%), Asians or Pacific Islanders (14%), adenocarcinoma histology (59%) and were more likely to present with distant metastases (68%). The young had better all cause and lung cancer-specific survival than the older patients (median survival for localized, regional, and distant disease: not reached, 28, 9 vs. 46, 17, 5 months; *p* < 0.001 for all groups). Male sex, non-adenocarcinoma histology, and main bronchial primary were independent negative prognostic factors among the young. In contrast to the overall population, black race was a poor prognostic factor among the young.

**Conclusion:**

The incidence of NSCLC in the young decreased from 1978 to 2010. The clinical characteristics of NSCLC in the young, including demographic distribution, treatment, and outcomes are different from those observed in the older patients.

## Introduction

Lung cancer is the most common cause of cancer-related death in the United States and worldwide (1, 2). Despite recent advances in treatment, prognosis of patients with lung cancer remains poor, with 5-year overall survival of approximately 15% (3). Non-small cell lung cancer (NSCLC), which accounts for over 85% of all lung cancer, is often considered a disease of the older population with a median age at diagnosis of about 70 years (3).

However, a significant proportion of new NSCLC patients, ranging between 1 and 10%, are younger than 40 years (4–9). There are several issues, which are particularly relevant to NSCLC in these patients, for example, their distinctive cancer biology, treatment tolerance, adherence, effectiveness, fertility preservation, and early death (10). Despite being an important demographic subgroup, there are limited data on the incidence, time-trends, and clinical characteristics of young patients with lung cancer.

Although there are substantial variability in the age ranges used in past studies, available data suggest that patients younger than 40 have a higher incidence of adenocarcinoma and greater representation of females (5, 7, 11–24). Reports comparing stage at presentation and outcomes of NSCLC in the young to older patients have yielded discordant results (11, 12, 14–16, 18, 20–22, 24).

Only two large population-based studies have studied lung cancer in the young using the surveillance, epidemiology, and end results (SEER) (5, 6). Since the recent report, there have been significant advances in the screening, diagnosis, and management of NSCLC. Additional registries have been included in SEER and the introduction of the normalized localized/regional/distant staging system have made comparison across a longer period more accurate compared with the American Joint Committee on Cancer staging used in prior studies. Furthermore, the population younger than 40 in itself is heterogeneous and previous studies have not explored clinicopathological features and outcomes among the different age groups constituting this larger group. In this study, using the normalized localized/regional/distant staging system, we analyzed the SEER database for NSCLC patients from 1998 to 2010 and evaluated characteristics of patients younger than 40. Using the data from 1975 to 2010, we also describe time-trends in the incidence of NSCLC in this population.

## Materials and Methods

SEER*Stat version 8.0.4 was used for all data collection and frequency analysis. For frequency analysis, SEER 18 Register Research Data+ Hurricane Katrina impacted Lousiana Cases, November 2012 sub (2973–2010 varying), released April 2013 were used to evaluate demographic characteristics and treatment received. Patient inclusion criteria are Site recode International Classification of Diseases for Oncology, third Edition 2008 Lung and Bronchus (old code C340–C349), and year of diagnosis 1998–2010, and NSCLC. NSCLC histology was categorized using International Classification of Diseases for Oncology, third Edition histology codes for malignant cases with the following groups: adenocarcinoma (8140, 8141, 8143, 8147, 8250-8255, 8260, 8310, 8430, 8480, 8481, 8490, 8570, 8571, 8572, 8573, 8574), squamous cell carcinoma (8052, 8070–8078), adenosquamous cell carcinoma (8560), large cell carcinoma (8012, 8013), and carcinoma not otherwise specified (NOS) (8010, 8046, 8050, 8051, 8575), undifferentiated (8020). Only malignant behavior, known age, case in research database cases was selected. Statistical homogeneity of the frequency distributions was examined using Chi-squared tests.

For survival time analysis, the same data registry and selection criteria were applied. Five-year survival rate and median survival for each patient subgroup were calculated using SEER STAT and R. 354,513 individual records were collected to generate Kaplan–Meier curves as well as hazard ratio (HR) estimates based on Cox proportional hazard models adjusted for multiple covariates (multivariate analysis) or each single covariates of concern (Univariate analysis). Kaplan–Meier curves were plotted using GraphPad Prism 6.0. Cox model fitting was done using R.

For analysis on the incidence and its trend, we used data from all nine registries covering 1975–2010. The estimated incidence rates were adjusted for patient age. Percentage change, annual percentage change were reported using SEERT STAT based on the method described previously (25) with modification for confidence intervals (CIs). Percentage change was calculated using data collected at 1-year intervals. APC was calculated using weighted least squares. Comparison of the incidence observed from two periods of 1975–1982 and 1983–2010 were reported.

## Results

### Time-trends in incidence of NSCLC in the young

**Figure 1:**
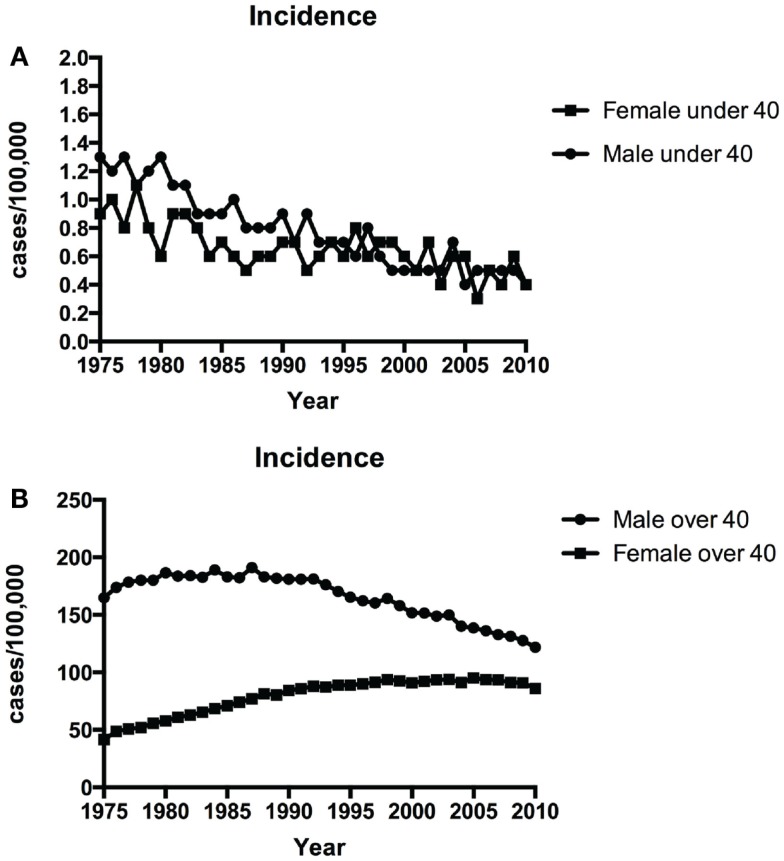
**Time-trends in incidence of NSCLC over time among patients <40 years (A) and ≥40 years (B) over 1975–2010**.

Figure 1A shows the time-trends in incidence of NSCLC in patients younger than 40 from 1975 to 2010. The incidence of NSCLC decreased in the young during this period: 1.1/100,000 in 1975 vs. 0.4/100, 000 in 2010 in males and 0.9/100,000 in 1975 vs. 0.4/100, 000 in 2010 in females. The annual percentage change in period 1983–2010 was statistically significant. Although NSCLC incidence in older males (over 40 years old) has declined over the same period (164.9/100, 000 in 1975 vs. 121.8/100,000 in 2010), the incidence in older females has increased (44.4/100,000 in 1975 vs. 86/100, 000 in 2010) (Figure 1B).

### Characteristics of NSCLC in the young

Table 1 shows the characteristics of NSCLC in patients younger than 40 compared with older patients. NSCLC still overwhelmingly remains a disease of older individuals: 99.4% of incident NSCLC in the United States between 1998 and 2010 were in patients older than 40. However, the male-predominant distribution of NSCLC in adults was compromised in the young among whom women had a statistically non-significantly higher incidence of NSCLC than men (51 vs. 49%; *p* = 0.14). Asians or Pacific Islanders accounted for 14% of young NSCLC whereas they accounted for only 5% among the older population. Although less marked, the frequency of Blacks was higher among those younger than 40 (15 vs. 11%). Adenocarcinoma and squamous cell lung cancer were the most common histologies among the young. The young had a higher frequency of adenocarcinoma (59 vs. 42%) and lower frequency of squamous lung cancers (10 vs. 24%) than the older population. Distant metastases at diagnosis were more common among the young (68 vs. 52%). Upper lobe cancers, which are thought to be associated with cigarette smoking, were less common among the young (44 vs. 50%). Thus, significant differences in frequency were observed in the young compared with older patients in terms of gender, race, histology, distant metastasis, and primary site of NSCLC.

**Table 1:** **Characteristics of NSCLC patients diagnosed between 1998 and 2010 by age group**.

	0–39 years		>40 years		*p* Value
	Count	Percentage	Count	Percentage	
**Total**	2786		458,217		
**Gender**					<0.0001
Men	1354	48.60%	253,324	55.28%	
Women	1432	51.40%	204,893	44.72%	
**Race**					<0.0001
White	1938	69.56%	379,694	82.86%	
Black	419	15.04%	51,337	11.20%	
American-Indian/Alaska Native	18	0.65%	1837	0.40%	
Asian or Pacific Islander	401	14.39%	24,497	5.35%	
Unknown	10	0.36%	852	0.19%	
**Histology**					<0.0001
Adenocarcinoma	1648	59.15%	194,374	42.42%	
Squamous	284	10.19%	111,467	24.33%	
Adenosquamous	45	1.62%	5,922	1.29%	
Large cell	165	5.92%	20,055	4.38%	
Carcinoma, NOS	638	22.90%	125,701	27.43%	
Undifferentiated	6	0.22%	698	0.15%	
**Staging**					<0.0001
*In situ*	0	0.00%	0	0.00%	
Localized	274	9.83%	86,388	18.85%	
Regional	490	17.59%	107,925	23.55%	
Distant	1902	68.27%	237,815	51.90%	
Unknown	120	4.31%	26,089	5.69%	
**Primary site**					<0.0001
Main bronchus	151	5.42%	19,593	4.28%	
Upper lobe	1228	44.08%	229,961	50.19%	
Middle lobe	139	4.99%	18,578	4.05%	
Lower lobe	620	22.25%	113,176	24.70%	
Overlapping lesion of lung	61	2.19%	6,002	1.31%	
Lung, NOS	587	21.07%	70,907	15.47%	

Although age by itself is not a major determinant of treatment for NSCLC, co-morbidities may limit the ability to deliver therapy. SEER does not provide sufficient data to estimate all treatment, but has information on cancer-directed surgeries. For all stages, the young were significantly more likely to undergo surgery than older patients (*p* = 0.0054 to <0.0001) (data not shown).

To better understand the clinicopathological characteristics of the young, we assessed them in 5-year age increments starting from age 15 (Table 2). Although individual age subgroups had limited number of patients, the results show significant differences in race, histology, stage at diagnosis, and primary site of involvement between the individual age groups.

**Table 2:** **Characteristics by age groups for patients <40 years (diagnosed between 1998 and 2010)**.

	15–19 years		20–24 years		25–29 years		30–34 years		35–39 years		*p* Value
	Count	Percentage	Count	Percentage	Count	Percentage	Count	Percentage	Count	Percentage	
**Gender**											0.8061
Male	10	45.45%	41	50.62%	101	50.75%	299	50.25%	895	47.89%	
Female	12	54.55%	40	49.38%	98	49.25%	296	49.75%	974	52.11%	
**Race**											0.0011
White	17	77.27%	64	79.01%	131	65.83%	388	65.21%	1325	70.89%	
Black	4	18.18%	8	9.88%	22	11.06%	94	15.80%	289	15.46%	
American-Indian/Alaska Native	0	0.00%	1	1.23%	0	0.00%	2	0.34%	14	0.75%	
Asian or Pacific Islander	1	4.55%	7	8.64%	46	23.12%	107	17.98%	236	12.63%	
Unknown	0	0.00%	1	1.23%	0	0.00%	4	0.67%	5	0.27%	
**Histology**											
Adenocarcinoma	16	72.73%	52	64.20%	121	60.80%	384	64.54%	1058	56.61%	0.021
Squamous cell carcinoma	4	18.18%	15	18.52%	21	10.55%	48	8.07%	195	10.43%	
Adenosquamous	0	0.00%	0	0.00%	4	2.01%	9	1.51%	32	1.71%	
Large cell carcinoma	0	0.00%	3	3.70%	13	6.53%	25	4.20%	124	6.63%	
Carcinoma, NOS	2	9.09%	11	13.58%	39	19.60%	129	21.68%	456	24.40%	
Undifferentiated	0	0.00%	0	0.00%	1	0.50%	0	0.00%	4	0.21%	
**Staging**											
*In situ*	0	0	0	0	0	0	0	0	0	0	<0.0001
Localized	10	45.45%	20	24.69%	25	12.56%	59	9.92%	155	8.29%	
Regional	2	9.09%	13	16.05%	27	12.56%	94	15.80%	348	12.13%	
Distant	10	45.45%	42	51.85%	134	67.34%	415	69.75%	1293	45.07%	
Unknown/unstaged	0	0.00%	6	7.41%	13	6.53%	27	4.54%	73	2.54%	
**Primary site**											
Main bronchus	1	4.55%	9	11.11%	9	4.52%	29	4.87%	98	5.24%	<0.0001
Upper lobe	8	36.36%	18	22.22%	56	28.14%	242	40.67%	901	48.21%	
Middle lobe	3	13.64%	9	11.11%	9	4.52%	35	5.88%	81	4.33%	
Lower lobe	7	31.82%	20	24.69%	54	27.14%	147	24.71%	387	20.71%	
Overlapping lesion of lung	1	4.55%	5	6.17%	8	4.02%	12	2.02%	34	1.82%	
Lung, NOS	2	9.09%	20	24.69%	63	31.66%	130	21.85%	368	19.69%	
**Number of primaries**											0.7131
One primary only	21	95.45%	74	91.36%	182	91.46%	529	88.91%	1674	89.57%	
Multiple primaries	1	4.55%	7	8.64%	17	8.54%	66	11.09%	195	10.43%	

### Outcomes of NSCLC in the young

**Figure 2:**
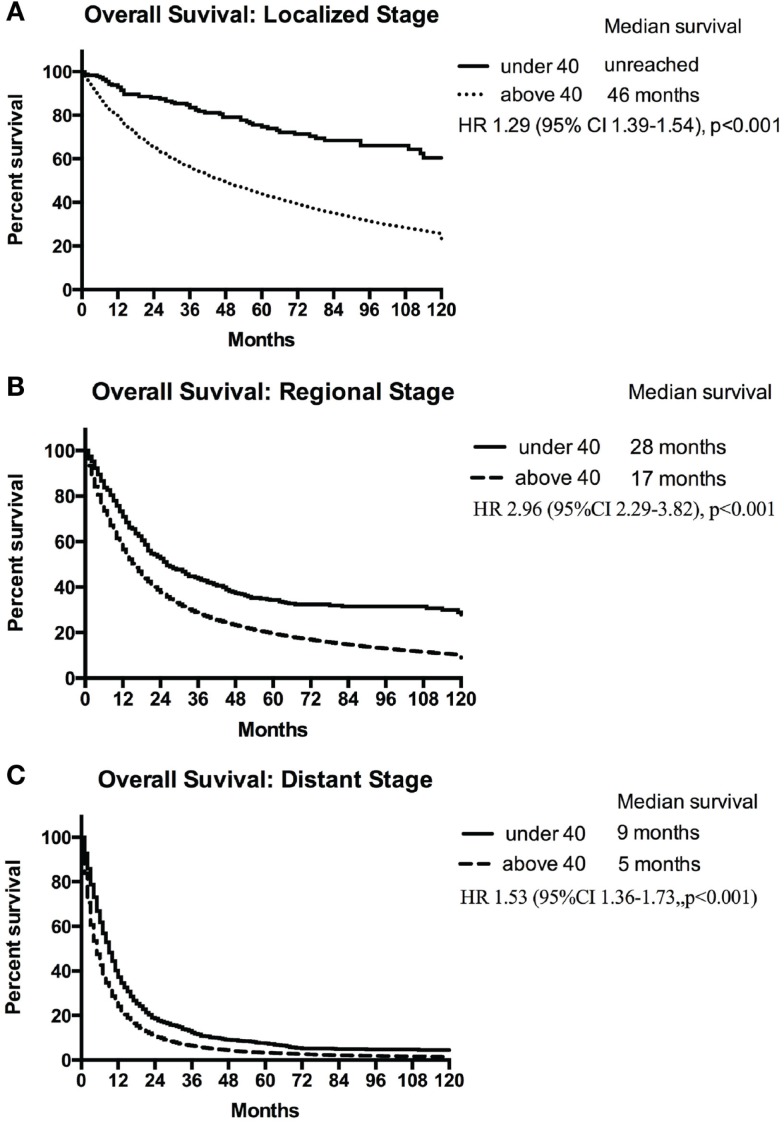
**Median survival of patients <40 years and ≥40 years with localized (A), regional (B), and distant (C) NSCLC**.

Using the standardized localized/regional/distant staging system, stage-for-stage, the young had superior overall survival than the older population. The differences in survival were more marked for localized and regional disease than for distant disease. Median survival of older population with localized disease was 46 months compared with the young for whom median survival was not reached (HR, 1.29; 95% CI 1.39–1.54; *p* < 0.001) (Figure 2A). Patients younger and older than 40 with regional disease had median survival of 28 and 17 months, respectively (HR, 2.96; 95% CI 2.29–3.82; *p* < 0.001) (Figure 2B). Median survival of the young and older patients with distant disease was 9 and 5 months, respectively (HR, 1.53; 95% CI 1.36–1.73; *p* < 0.001) (Figure 2C). The young also had a lower lung cancer-specific death rate than their older counter parts. The 5-year lung cancer specific survival for under 40 vs. over 40 age groups were 91.2 vs. 55.9% for localized, 50.7 vs. 26.7% for regional, 9.7 vs. 4.2% at distant stage (Table 3).

**Table 3:** **Lung cancer specific survival for patients under and over 40**.

	5-year survival (%)*	CI 95%	*p* Value
**Localized**			<0.001
<40	91.2	88.7–93.2	
≥40	55.9	55.4–56.3	
**Regional**			<0.001
<40	50.7	46.6–54.6	
≥40	26.7	26.3–27.0	
**Distant**			<0.001
<40	9.7	8.2–11.3	
≥40	4.2	4.1–4.3	

*Cause-specific survival: non-lung cancer death counted as alive/sensored.

Based on the Cox proportional hazard model adjusted for multiple covariates, the risk of death was higher for those older than 40 (HR, 1.45; 95% CI, 1.39–1.52; *p* < 0.001). Other independent negative prognostic factors included male sex (HR, 1.16; 95% CI, 1.15–1.17; *p* < 0.001), non-adenocarcinoma histology, and location of primary tumor in the main bronchus, middle lobe, or upper lobe. Favorable prognostic factors included Asian/Pacific Islander (HR, 0.78; 95% CI, 0.765–0.790; *p* < 0.001) or Black race (HR 0.98; 95% CI 0.969–0.991; *p* = 0.0004), localized (HR, 0.38; 95% CI 0.378–0.388; *p* < 0.001), or regional disease (HR, 0.54; 95% CI 0.536–0.547; *p* < 0.001), upper lobe primary, surgical resection, and year of diagnosis 2004–2010 compared with 1998–2003.

Among those younger than 40 (Table 4), the risk of death was higher for males compared with females (11 vs. 15 months; HR, 1.174; 95% CI, 1.071–1.285; *p* < 0.001). As in the overall population, Asian/Pacific Islander race was an independent positive prognostic factor (HR, 0.831; 95% CI 0.727–0.950). In contrast to the overall population, Black race was a poor prognostic factor among the young (HR, 1.22; 95% CI, 1.075–1.384; *p* < 0.001). Independent negative prognostic factors included non-adenocarcinoma histology and main bronchial primary (HR 1.23; 95% CI, 1.009–1.507). Surgical resection (HR, 0.497; 95% CI, 0.434–0.570) and the year of diagnosis 2004–2010 compared with 1998–2003 (HR, 0.746; 95% CI 0.678–0.821) were other favorable prognostic factors.

**Table 4:** **Multivariate analysis to examine potential factors associated with survival in the young**.

	*N*	Median survival	1-year survival rate (95% CI)	5-year survival rate (95% CI)	HR (univariate analysis)	*p* Value	HR (multivariate)	*p* Value
**Gender**
Men	1271	11	43.9 (41.2, 46.8)	18.0 (15.8, 20.6)	1.22 (1.115, 1.335)	1.56E-05	1.174 (1.071, 1.285)	0.000566
Women	1276	15	54.9 (52.1, 57.7)	21.2 (18.8, 23.8)	1		1	
**Race**
White	1759	12	49.3 (46.9, 51.7)	20.7 (18.7, 22.9)	1		1	
Black	383	10	41.8 (37.1, 47.2)	15.5 (12.0, 20.0)	1.193 (1.054, 1.351)	0.005	1.220 (1.075, 1.384)	0.00199
American-Indian/Alaska Native	17	8	41.2 (23.3, 72.7)	29.4 (14.1, 61.4)	0.947 (0.536, 1.672)	0.852	1.214 (0.684, 2.154)	0.508
Asian or Pacific Islander	378	15	57.6 (52.7, 63.0)	15.8 (11.8, 21.3)	0.950 (0.833, 1.084)	0.446	0.831 (0.727, 0.950)	0.0065
Unknown	10	N/A	N/A: only 2 events	N/A	0.178 (0.045, 0.713)	0.015	0.245 (0.061, 0.986)	0.0477
**Histology**
Adenocarcinoma	1524	15	55.0 (52.5, 57.7)	21.3 (19.0, 23.8)	1		1	
Squamous	244	12	47.8 (41.8, 54.7)	22.5 (17.5, 28.9)	1.065 (0.911, 1.246)	0.428	1.144 (0.973, 1.345)	0.1028
Adenosquamous	41	10	49.0 (35.5, 67.5)	12.9 (5.69, 29.2)	1.306 (0.929, 1.838)	0.125	1.833 (1.299, 2.589)	0.000572
Large cell	154	9	34.3 (27.5, 42.8)	13.2 (8.64, 20.2)	1.398 (1.166, 1.678)	0.0003	1.332 (1.107, 1.603)	0.00243
Carcinoma, NOS	579	9	39.9 (36.0, 44.1)	16.4 (13.4, 20.0)	1.356 (1.216, 1.513)	4.56e-08	1.123 (1.003, 1.258)	0.0446
Undifferentiated	5	4	40.0 (13.67, 1)	N/A	2.528 (1.050, 6.087)	0.039	1.793 (0.742, 4.336)	0.1949
**Staging**
Localized	237	>114	92.9 (89.6, 96.3)	74.8 (69.6, 81.5)	0.114 (0.088, 0.148)	<2e-16	0.190 (0.144, 0.252)	<2e-16
Regional	434	28	73.5 (69.4, 77.9)	34.2 (29.6, 39.5)	0.369 (0.323, 0.422)	<2e-16	0.496 (0.427, 0.576)	<2e-16
Distant	1777	9	37.2 (34.9, 39.6)	7.53 (6.20, 9.15)	1		1	
Unknown	99	17	59.0 (49.9, 69.8)	35.4 (26.6, 47.2)	0.439 (0.340, 0.569)	3.92E-10	0.368 (0.281, 0.481)	2.38 e-13
**Primary site**
Main bronchus	142	8	29.3 (22.6, 38.0)	18.1 (12.5, 26.4)	1.477 (1.215, 1.797)	9.43E-05	1.233 (1.009, 1.507)	0.0411
Upper lobe	1130	13	51.3 (48.4, 54.4)	21.6 (19.1, 24.4)	1		1	
Middle lobe	129	18	62.7 (54.7, 71.9)	21.7 (15.1, 31.4)	0.862 (0.695, 1.067)	0.173	0.965 (0.778, 1.196)	0.7423
Lower lobe	564	16	56.4 (52.4, 60.8)	21.7 (18.0, 26.0)	0.899 (0.797, 1.013)	0.081	0.922 (0.817, 1.041)	0.1902
Overlapping lesion of lung	52	14	53.1 (40.3, 69.8)	19.4 (10.3, 36.9)	1.040 (0.745, 1.452)	0.817	0.968 (0.692, 1.353)	0.8489
Lung, NOS	530	10	39.8 (35.7, 44.4)	12.9 (10.0, 16.7)	1.342 (1.194, 1.509)	8.41E-07	0.999 (0.886, 1.128)	0.994
**Surgery performed or not**
Yes	729	46	77.2 (74.1, 80.3)	45.1 (41.3, 49.3)	0.301 (0.268, 0.338)	<2e-16	0.497 (0.434, 0.570)	<2e-16
No	1779	9	37.9 (35.6, 40.3)	8.45 (7.05, 10.12)	1		1	
Unknown	39	10	46.2 (32.3, 66.0)	24.6 (13.5, 44.8)	0.624 (0.423, 0.920)	0.0171	0.986 (0.664, 1.463)	0.943
**Year of diagnosis**
1998–2004	1416	11	45.1 (42.6, 47.8)	17.6 (15.7, 19.8)	1		1	
2004–2010	1131	15	55.4 (52.4, 58.5)	22.2 (19.1, 25.9)	0.822 (0.748, 0.902)	4.08E-05	0.746 (0.678, 0.821)	2.16 E-09

## Discussion

Non-small cell lung cancer overwhelmingly remains a disease of the older population. A smaller, but not insignificant proportion of patients with NSCLC are younger than 40. The epidemiology of NSCLC in the young and their clinical characteristics are not well defined. Based on these considerations, we used the SEER database to conduct a large population-based study of NSCLC in the young. Our aims were to evaluate the time-trends in incidence, clinicopathologic characteristics, and prognostic factors of NSCLC in the young.

Young patients represented 0.6% of incident NSCLC from 1978 to 2010. The incidence of young NSCLC declined significantly during this time. Young NSCLCs had a higher proportion of women, Asians or Pacific Islanders, adenocarcinoma histology and were more likely to present with distant metastases at diagnosis than the older population. The young were less likely to have upper lobe cancers.

Although for purposes of observational studies, patients younger than 40 are grouped into one category, we found that this group in itself was heterogeneous in terms of clinical and pathological characteristics. Within the young population, significant differences were observed in the frequency of race, histology, stage at diagnosis, and primary site of involvement. Using the standardized localized/regional/distant staging system, stage-for-stage, the young had better all cause and lung cancer-specific survival than the older population. Multivariate Cox model analyses identified male sex, non-adenocarcinoma histology, and main bronchial primary as independent negative prognostic factors among the young. Additionally, in contrast to the overall population, black race was a poor prognostic factor among the young.

Our results extend upon those from prior population-based registry studies, which showed a higher proportion of women, adenocarcinoma, and metastatic disease in the young (5, 6). As in our study, both prior studies found somewhat modest increases in the overall magnitude of risk of death. In addition to the previous studies, we show a decrease in the incidence of NSCLC from 1975 to 2010 and demonstrate significant differences in age-based subgroups among those younger than 40.

Cancer rates in the young are thought to be a useful indicator to assess the impact of recent cancer control efforts. Using population-based registries with state-specific data, Jemal et al. have demonstrated that lung cancer death rates in young adults aged between 30 and 39 years correlated strongly and inversely with the individual state tobacco control efforts (26). The observed decline in the NSCLC death rates among the young in our study could therefore be attributed in part to the past anti-smoking interventions (27).

Our finding of a comparable incidence of NSCLC among males and females in the young indicates the effect of factors other than cigarette smoking. This is in contrast to older population wherein smoking is the major risk factor for lung cancer. In them, the convergence of relative risks of NSCLC for men and women have been attributable to the convergence of smoking patterns among men and women since 1960s and the aging of birth cohorts with the heaviest lifetime history of smoking (28). Despite a probable early start of smoking in young adults with lung cancer, the development of the disease cannot be fully explained due to smoking alone. Several studies have indicated higher genetic susceptibility among younger patients (29–31), as well as influence of occupational risk factors (32). There are limited data on the genetic basis of lung cancer susceptibility (33). Recent studies have reported that germline mutations in *EGFR* and *HER2* genes can predispose the development of lung cancer (34, 35).

We were unable to clarify the incidence of smoking in the young NSCLC since smoking status is not captured in the SEER database. Nevertheless, a growing body of literature suggests that NSCLC in never-smokers has several distinct clinical, pathological, and molecular characteristics implying that it might be a different disease compared with NSCLC in smokers (36); adenocarcinoma is the most frequent histology in never-smokers and alterations in driver oncogenes such as *EGFR*, and *ALK* are more common in tumors from never-smokers compared to smokers.

Although based on a relatively small number of patients, our findings indicate that black race was a poor prognostic factor among the young. Whether this disparity is related to genetic differences in the tumor, pharmacogenomics, or differential access to care will need additional study. Previous studies have suggested that patients with upper lobe tumors tend to have had more exposure to tobacco. In our study, upper lobe cancers were less common among the young, further supporting the notion that genetic susceptibility and other environmental factors may be important risk factors in the pathogenesis of NSCLC in the young (37).

This study provides the most comprehensive analyses to date of a large registry dataset of young NSCLC. We observed significant differences in clinical presentation even among those younger than 40. Further studies are needed to understand the interaction between genetic susceptibility and environmental carcinogens including tobacco in the pathogenesis of NSCLC in the young.

## Author Contributions

AT, YC, MJ, and GG designed the work. YC and TY performed the data acquisition and analysis. AT, YC, MJ, TY, and GG did the interpretation. AT, YC, and MJ drafted the manuscript. All authors approved the final version of the manuscript.

## Conflict of Interest Statement

The authors declare that the research was conducted in the absence of any commercial or financial relationships that could be construed as a potential conflict of interest.
